# Comparative analysis of postoperative curative effect of liver wedge resection and liver IVb + V segment resection in patients with T2b gallbladder cancer

**DOI:** 10.3389/fsurg.2023.1139947

**Published:** 2023-03-17

**Authors:** Zichao Wang, Hongliang Liu, Yong Huang, Jun Wang, Junjie Li, Lingpeng Liu, Mingwen Huang

**Affiliations:** Department of General Surgery, the Second Affiliated Hospital of Nanchang University, Nanchang, China

**Keywords:** gallbladder cancer, operation methodes, curative effect, t2b, analysis

## Abstract

**Objective:**

To compare the therapeutic effects of wedge resection and liver segment IVb + V resection on patients with T2b gallbladder cancer.

**Methods:**

The clinical and pathological data of 40 patients with gallbladder cancer admitted to the Second Affiliated Hospital of Nanchang University from January 2017 to November 2019 were retrospectively analyzed, and they were divided into two groups according to different surgical methods. The control group underwent liver wedge resection, while the experimental group underwent liver segment IVb + V resection. Preoperative age, bilirubin index, tumor markers, postoperative complications and survival were compared between the two groups. Log-rank test was used for univariate analysis, and Cox proportional hazards regression model was used for multivariate analysis. Kaplan-Meier survival curves were drawn.

**Results:**

Univariate analysis showed that tumor markers and degree of differentiation were the risk factors for the prognosis of patients with gallbladder carcinoma after radical cholecystectomy (*P* < 0.05). Multivariate analysis showed that the elevation of CA125 and CA199, poor differentiation, and Lymph node metastasis were independent risk factors for the prognosis of gallbladder carcinoma after radical resection (*P* < 0.05). According to the 3-year survival rate, the survival rate of patients with liver 4B + 5 segment resection + cholecystectomy was higher than that of patients with 2 cm liver wedge resection + cholecystectomy (41.6% vs. 72.7%).

**Conclusion:**

Patients with T2b gallbladder cancer should be treated with liver segment IVb + V resection, which is helpful to improve the prognosis of patients and should be widely promoted.

Gallbladder cancer (carcinoma of gallbladder) is one of the most common gallbladder malignant tumors and one of the most common biliary tract malignant tumors. Because gallbladder cancer has no specific symptoms in the early stage, patients often undergo cholecystectomy for benign gallbladder diseases, and gallbladder cancer is diagnosed by pathological examination after operation. When the right upper abdominal mass can be palpated, it is often in the late stage, accompanied by jaundice, ascites and systemic symptoms (1). Because gallbladder cancer is mostly ineffective to chemotherapy and radiotherapy, surgical resection is often the best treatment, and the scope of surgical resection is determined according to the stage of gallbladder cancer (2–4). In TNM staging of gallbladder carcinoma (8th edition of AJCC), tumors on the liver side that invade the perimuscular connective tissue and do not extend beyond the serosa are classified as stage T2b. At present, there is no consensus on the surgical treatment of T2b gallbladder cancer at home and abroad, which is generally wedge resection of the liver (2 cm wedge resection of the liver along the edge of the gallbladder bed according to the extent of the lesion) or liver segment IVb + V resection. The purpose of this article is to explore the impact of different surgical methods on the prognosis of patients with T2b gallbladder cancer.

## 1. Objects and methods

### 1.1. General information

A total of 40 patients with gallbladder cancer were selected from the Second Affiliated Hospital of Nanchang University from January 2017 to November 2019. There were 17 males and 23 females, the ratio of male to female was 9:13, the age ranged from 45 to 80 years, and the median age was 66 years. Inclusion criteria: (1) Gallbladder carcinoma was confirmed by pathological examination and complete preoperative imaging data; (2) TNM staging was T2b, and tumor staging was AJCC version 8; (3) Complete clinical data were available for analysis and evaluation. Exclusion criteria: (1) gallbladder cancer with other tumors; (2) preoperative neoadjuvant therapy; (3) palliative treatment; (4) incomplete data.

### 1.2. Preoperative auxiliary examination laboratory tests

Included blood routine, biochemical indicators and tumor markers. Imaging data included abdominal ultrasound and abdominal CT to confirm the diagnosis.

### 1.3. Operation method

Wedge resection of liver was performed in the control group, including cholecystectomy and wedge resection of 2 cm at the edge of gallbladder bed. Cholecystectomy and liver IVb + V segmentectomy were performed in the experimental group. The scope of standard regional lymph node dissection: the neck of gallbladder, common bile duct, portal vein and proper hepatic artery lymph nodes; the scope of extended regional lymph node dissection: the scope of conventional lymph node dissection + lymph nodes around the common hepatic artery, regional lymph nodes behind the head of pancreas and other more distant lymph nodes.

### 1.4. Analysis indicators

The preoperative clinical sign and symptom, preoperative laboratory examination results, surgical conditions, postoperative pathological data, types and time of postoperative complications were analyzed. Demographic characteristics (gender, age), preoperative bilirubin index, preoperative tumor markers, pathological data (tumor type, differentiation degree, liver invasion, nerve invasion, lymph node metastasis), postoperative complications (hypoproteinemia, ascites, pleural effusion, abdominal infection) were included in univariate and multivariate analysis.

### 1.5. Follow-up

We used outpatient follow-up and telephone follow-up. Each patient was required to return to the hospital for CT examination and detection of tumor markers and biochemical indicators in January, March and June after the operation. Telephone follow-up was conducted every 3–6 months after the sixth month after the operation. The survival time of the patient was recorded with the death of the patient as the end point. The follow-up time was up to July 1, 2021. The follow-up showed that the survival time was 4–36 months, 15 cases were still alive, 4 cases were lost to follow-up, the loss rate was 10%.

### 1.6. Statistical analysis

SPSS 25.0 statistical software was used to analyze the results. Measurement data conforming to normal distribution are ($\bar{X}\pm S$), M (range) for measurement data of skew distribution, and percentage for count data. Log-rank test was used for univariate analysis, and Cox proportional hazards regression model was used for multivariate analysis. *P* < 0.05 indicates statistical significance. Kaplan-Meier survival curves were drawn.

## 2. Result

### 2.1. General clinical manifestations

We collected 40 patients with primary gallbladder cancer admitted to the Second Affiliated Hospital of Nanchang University from January 2017 to January 2019, including 20 cases of accidental gallbladder cancer. Among the 40 patients, 9 cases were asymptomatic, 31 cases had distending pain in the right upper abdomen, 21 cases had radiating pain in the waist and back, and 9 cases had fever. There were 32 patients with gallstones, 8 patients with gallbladder polyps, 12 patients with hypertension and 17 patients with diabetes mellitus.

### 2.2. Preoperative laboratory examination items

Preoperative examination of tumor markers is the main method to assist diagnosis, and the elevation of CA199 and CA125 is considered to have certain clinical value in clinical work. Among the 40 patients, there were 11 patients whose tumor markers were normal. There were 12 patients (45.5%) with elevated CA199, 3 patients (20.5%) with elevated CA125, and 14 patients (34.1%) with CA199 and CA125.

### 2.3. Operation-related conditions and postoperative complications

All patients underwent radical cholecystectomy and open cholecystectomy. The surgeons were all experienced doctors in the Department of Hepatobiliary and Pancreatic Surgery of the Second Affiliated Hospital of Nanchang University. In the observation group, the gallbladder and the liver tissue 2 cm deep in the gallbladder bed were resected together. In the control group, after cholecystectomy, the hepatogastric ligament was opened, and a blocking band was placed in the hepatoduodenal ligament. After the beginning of blocking, the liver parenchyma was cut off from the visceral surface (routine blocking for 15 min and releasing for 5 min), and the IVb and V segments were clipped after exposing the hepatic pedicle. The ultrasonic scalpel was used to cut off the liver along the ischemic line until it was completely cut off. After the liver was cut, the abdomen was closed after it was clear that there was no oozing of blood on the wound surface. Patients in both groups were closely followed up after operation, and standard regional lymph node dissection was performed in both groups during the operation. Extended regional lymph node dissection was performed in patients with suspected metastasis based on preoperative imaging data or patients with regional lymph node enlargement confirmed by frozen biopsy during operation. Postoperative pathology showed regional lymph node metastasis in 13 patients, including 5 cases of liver metastasis and 2 cases of distant lymph node metastasis. Perioperative complications occurred in 11 patients, including pleural effusion in 7 cases, abdominal infection in 1 case, and liver dysfunction in 3 cases. There were no perioperative deaths.

### 2.4. Pathological examination results

There were 38 cases of adenocarcinoma, 1 case of squamous carcinoma and 1 case of mixed small cell neuroendocrine carcinoma. The degree of differentiation was low in 13 cases, moderate in 19 cases, high in 6 cases, and unknown in 2 cases.

### 2.5. Follow-up

**Figure 1:**
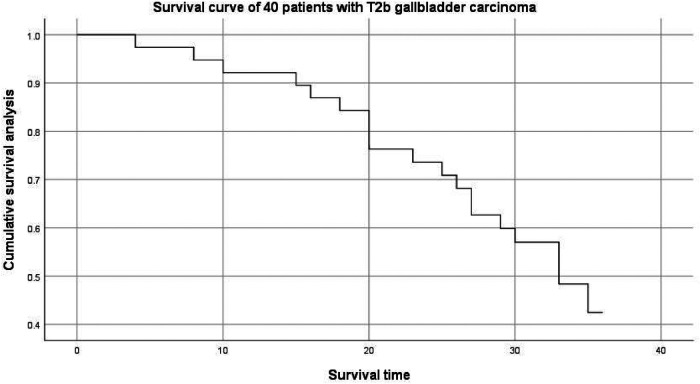
Survival curve of 40 patients with T2b gallbladder carcinoma.

The median follow-up time was 30 (4. 0–36. 0) months, and the median survival time was 29 (4. 0–36. 0) months. The overall survival curve of patients with gallbladder cancer collected in this study is shown in Figure 1.

### 2.6. Univariate analysis of prognostic factors

Univariate analysis showed that preoperative abnormal bilirubin (TBIL and DBIL increased), tumor markers (CA199 increased and CA125 increased), degree of differentiation (poor differentiation) and lymph node metastasis were the risk factors affecting the prognosis of gallbladder cancer after radical operation (*P* < 0.05). Age and preoperative bilirubin abnormalities are not important factors affecting the prognosis of radical cholecystectomy for gallbladder cancer (see Table 1 and Table 2). According to the 3-year survival rate, the survival rate of patients with liver 4B + 5 segment resection + cholecystectomy was higher than that of patients with 2 cm liver wedge resection + cholecystectomy.

**Table 1:** Univariate analysis of prognostic factors in patients with gallbladder cancer undergoing 2 cm wedge resection of liver and cholecystectomy^a^.

Clinical and pathological factors	Cases	3-year survival rate	*χ* ^2^	*P*
**Age**
>=60	13	30.8%	0.002	0.968
<60	10	30%		
**Preoperative bilirubin**
Abnormal (elevated TBIL and DBIL)	8	37.5%	16.85	0.049
Normal	15	26.7%		
**Tumor markers**
CA199 elevation	8	12.5%	17.682	0.01
CA125 elevation	2	50%		
CA125 and CA199 elevation	5	20%		
Normal	8	37.5%		
**Degree of differentiation** ^b^
High differentiation	3	66.7%	33.143	0.021
Medium differentiation	12	33.3%		
Low differentiation	8	12.5%		
**Lymph node metastasis**
Yes	6	16.7%	5.232	0.022
No	17	52.9%		
Postoperative complications	2	50%	0.367	0.545

^a^ Two cases were not included due to loss of follow-up.

^b^ One case was not included due to unknown degree of differentiation.

**Table 2:** Univariate analysis of prognostic factors in patients with gallbladder carcinoma undergoing liver 4B + 5 segment resection + cholecystectomy^a^.

Clinical and pathological factors	Cases	3-year survival rate	χ^2^	*P*
**Age**
>=60	5	40%	3.460	0.63
<60	8	37.5%		
**Preoperative bilirubin**
Abnormal (elevated TBIL and DBIL) 7	42.8%	2.356	0.045	
Normal	6	83.3%		
**Tumor markers**
CA199 elevation	0	none	6.993	0.030
CA125 elevation	1	100%		
CA125 and CA199 elevation	9	55%		
Normal	3	66.7%		
**Degree of differentiation** ^b^
High differentiation	3	66.7%	5.186	0.045
Medium differentiation	6	50%		
Low differentiation	6	33.3%		
**Lymph node metastasis**
Yes	6	66.7%	3.943	
No	7	14.3%		
Postoperative complications	6	33.3%	0.124	0.724

^a^ Two cases were not included due to loss of follow-up.

^b^ One case was not included due to unknown degree of differentiation.

### 2.7. Multivariate analysis of prognostic factors

The results of multivariate analysis showed that the elevation of CA125 and CA199 in tumor markers, poor pathological differentiation and lymph node metastasis were the independent risk factors affecting the prognosis of radical cholecystectomy for gallbladder cancer (*P* < 0.05) (Table 3 and Table 4).

**Table 3:** Multivariate analysis of prognostic factors in patients with gallbladder cancer undergoing 2 cm hepatic wedge resection plus cholecystectomy.

Clinical and pathological factors	B	SE	World	*P*	Exp (B)	CI of 95.0% Exp (B)	
						Lower Limit	Upper Limit
Preoperative bilirubin Abnormal (elevated TBIL and DBIL)/Normal	1.386	0.755	3.368	0.066	0.250	0.057	1.099
Tumor markers (CA125 elevation/CA199 elevation/CA125 and CA199 elevation)	0.880	0.459	3.672	0.045	2.412	0.980	5.935
Degree of differentiation (High differentiation/)	1.835	0.619	8.788	0.03	0.160	0.047	0.537
Lymph node metastasis (Yes/No)	2.468	1.138	3.713	0.03	11.798	1.269	109.729

Patients lost to follow-up are not included.

**Table 4:** Multivariate analysis of prognostic factors in patients with gallbladder carcinoma undergoing 4B + 5 segment hepatectomy + cholecystectomy.

Clinical and pathological factors	B	SE	World	*P*	Exp (B)	CI of 95.0% Exp (B)	
						Lower Limit	Upper Limit
Preoperative bilirubin Abnormal (elevated TBIL and DBIL)/Normal	0.916	0.758	1.463	0.226	4.000	0.910	17.582
Tumor markers (CA125 elevation/CA199 elevation/CA125 and CA199 elevation)	2.273	0.630	1.026	0.049	0.707	0.585	3.353
Degree of differentiation (High differentiation/)	0.230	0.333	0.477	0.490	0.795	0.414	1.525
Lymph node metastasis (Yes/No)	2.238	1.162	3.713	0.044	9.379	0.962	91.390

Patients lost to follow-up are not included.

## 3. Discussion

Because the clinical manifestations of early gallbladder cancer are not obvious, the disease often progresses or is accidentally found during cholecystectomy due to gallstones or polyps, and because there are differences in the surgical treatment of patients with T2b gallbladder cancer, radical cholecystectomy is generally considered to be the first choice for T2 gallbladder cancer. Simple cholecystectomy is associated with occult invasion or hepatic dissemination with an unknown risk of lymphatic spread (5–7). However, the optimal extent of hepatectomy based on the location of the primary tumor is still unclear, which has become a key issue in the current clinical study of gallbladder cancer. Because of the special anatomical characteristics of the gallbladder wall, there is no muscularis mucosa in the gallbladder wall, and the muscularis propria is a thin single layer, and there is no serosa in the side of the liver, only loose connective tissue between the gallbladder and the liver is connected with the Glission sheath of the liver. The metastatic pathways of gallbladder cancer mainly include lymphatic metastasis and hematogenous metastasis. Liver metastases are mainly hematogenous because there is no lymph flow from the gallbladder into the liver through the cystic duct. For the gallbladder vessels on the peritoneal side, there are usually 1–2 gallbladder veins that converge into the veins at the hepatic portal. The gallbladder vessels on the hepatic side usually have 2–20 gallbladder veins flowing directly into the hepatic portal system. Based on the large-scale study in the 8th edition of AJCC, the prognosis of T2b gallbladder cancer was reported to be poor. Liver recurrence has also been found to be common in stage T2 gallbladder cancer in a considerable part of the literature (6, 8).

Resection of liver segment IVb and V or resection of liver 2 cm along the edge of gallbladder bed in radical resection of gallbladder cancer is based on the theory that most of the gallbladder veins drain through the liver segment IVb and V. 2164;. However, invasion of the gallbladder vein by gallbladder carcinoma has been reported in only 0%–10% of all cases (6). Studies have found that resection of liver tissue combined with gallbladder cancer on the side of the liver can prolong the survival time of patients after operation (8). In this study, two methods of radical cholecystectomy were used for patients with T2b gallbladder cancer. Age, preoperative bilirubin changes, tumor markers changes, pathological differentiation, lymph node metastasis, postoperative complications and follow-up were compared, and survival time was recorded. The purpose was to provide a basis for the clinical treatment of T2b primary gallbladder cancer and benefit more patients.

**Figure 2.**
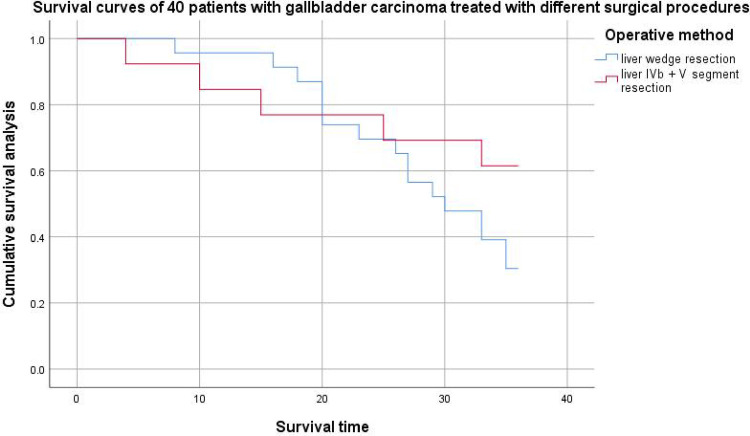
Survival of 40 patients gallbladder carcinoma treated with different surgical procedures

**Figure 3.**
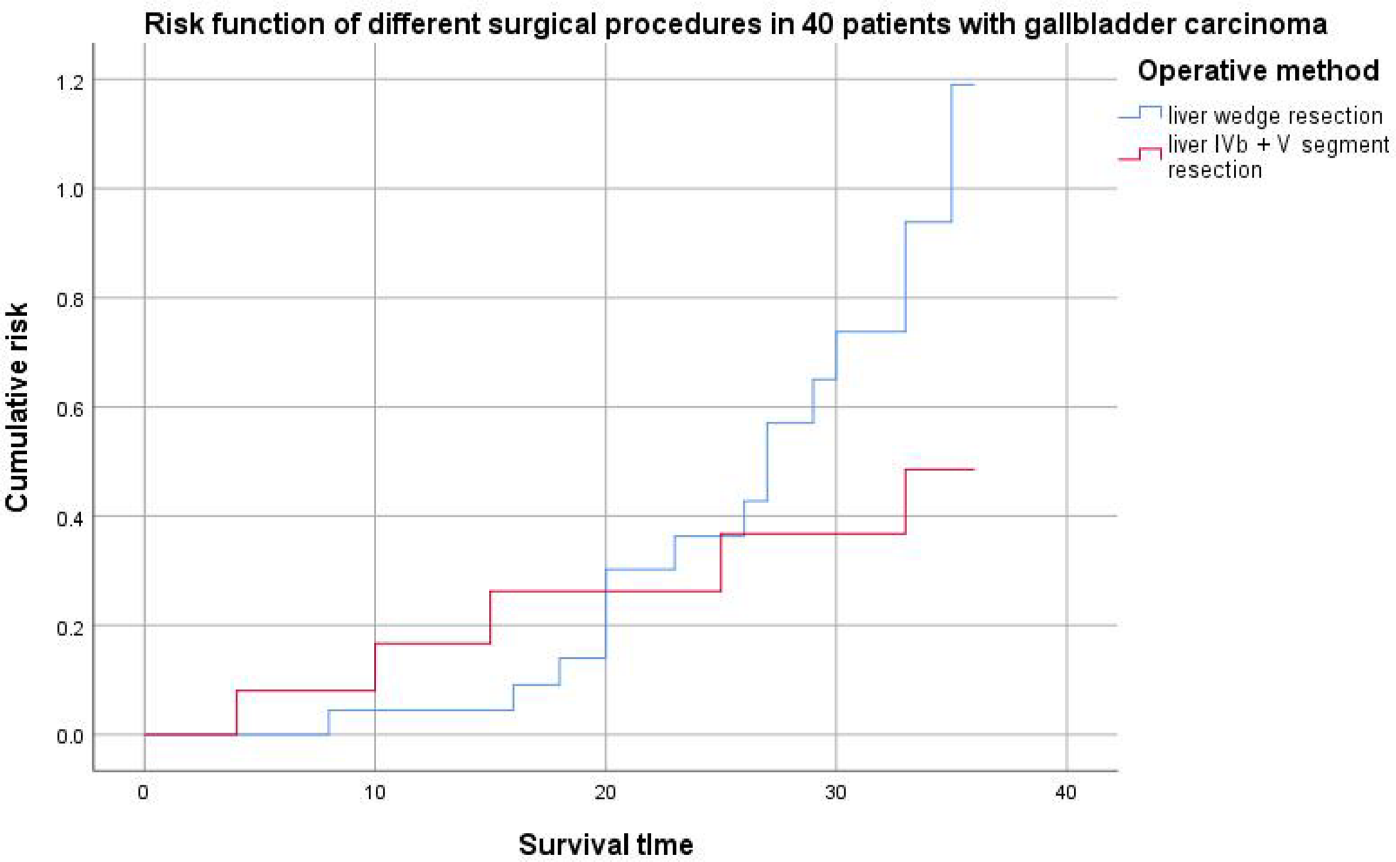
Risk function of surgical procedures in 40 patients with gallbladder carcinoma

The results of this study showed that preoperative abnormal bilirubin (TBIL and DBIL increased), tumor markers (CA199 increased and CA125 increased), differentiation (poorly differentiated) and lymph node metastasis were important factors affecting the prognosis of gallbladder cancer patients (*P* < 0.05). Age, preoperative bilirubin index and postoperative complications were not important factors affecting the prognosis of patients with gallbladder cancer (*P* > 0.05). According to the survival curve (Figure 2) and the 3-year survival rate, the short-term survival rate of patients with 2 cm liver wedge resection and cholecystectomy was higher than that of patients with liver segment IVb + V + cholecystectomy, but the 3-year survival rate was lower than that of patients with liver segment IVb + V + cholecystectomy. From the risk curve (Figure 3), the short-term cumulative risk of patients with 2 cm liver wedge resection + cholecystectomy was lower than that of patients with liver segment IVb + V + cholecystectomy, and with the prolongation of survival time, the short-term cumulative risk of patients with 2 cm liver wedge resection + cholecystic resection was gradually higher than that of the patients with liver segment IVb + V + cholecystic resection. This may be related to the advantage of less blood loss during the operation in patients with 2 cm wedge resection of liver and cholecystectomy, less damage to the patient's body during the operation, which is conducive to the recovery of patients after the operation, which can also be reflected in the number of postoperative complications. The operation time was longer in patients with liver segment IVb + segment V + cholecystectomy, and the amount of bleeding during the operation was more. The damage to the patient's body is relatively large, so there are more cases of postoperative complications. In addition, the probability of vascular and nerve invasion in patients with gallbladder cancer in stage T2b was much higher than that in stage T2a. In this stage, the range of gallbladder cancer cells flowing into the liver through the gallbladder vein was about 2 cm–5 cm from the gallbladder bed on average, and at least one direction was more than 4 cm. Therefore, from the point of view of improving the survival rate of patients, when patients with stage T2 gallbladder cancer, especially patients with stage T2b gallbladder cancer, liver segment IVb + V resection should be performed to achieve the therapeutic goal (9, 10).

Surgical treatment is still the most effective treatment for gallbladder cancer. For patients with stage T1b and above, current guidelines recommend extended radical resection, and extended resection of the tumor is beneficial to patient survival (11). Radical resection of gallbladder cancer is considered to be an important prognostic factor. According to Goetze et al. (12), in order to ensure R0 resection, resection of at least liver segment IVb + V should be performed for T2b gallbladder cancer. Surveys have shown that the survival rate of patients with simple cholecystectomy is low, and the five-year survival rate is 17%–20% (13). The most common recurrent types of T2b gallbladder cancer are para-aortic lymph node recurrence and intrahepatic metastasis (14), and T2b tumors have a better survival trend after radical cholecystectomy. It has been reported that the lymph nodes around the cystic duct, the common bile duct, the portal vein and the posterior superior lymph nodes of the pancreatic head are the lymph node groups with the highest probability of metastasis. Therefore, dissection of lymph nodes around the head of pancreas and common hepatic artery may be helpful to further improve the prognosis of patients with gallbladder cancer. Lymph node metastasis is a significant adverse prognostic factor, and adjuvant chemotherapy should be considered in patients with lymph node metastasis (15).

To sum up, this study shows that in order to achieve the goal of treatment, patients with T2b gallbladder cancer should be treated with liver segment IVb + V resection, which is helpful to improve the prognosis of patients and should be widely promoted. However, due to the small sample size and single-center retrospective study, the above conclusions still need to be widely verified.

## Data Availability

The raw data supporting the conclusions of this article will be made available by the authors, without undue reservation.
